# How to build and evaluate an integrated health care system for chronic patients: study design of a clustered randomised controlled trial in rural China

**DOI:** 10.5334/ijic.1846

**Published:** 2015-03-16

**Authors:** Wenxi Tang, Xiaowei Sun, Yan Zhang, Ting Ye, Liang Zhang

**Affiliations:** School of Medicine and Health Management, Huazhong University of Science and Technology, Wuhan, China; School of Medicine and Health Management, Huazhong University of Science and Technology, Wuhan, China; School of Medicine and Health Management, Huazhong University of Science and Technology, Wuhan, China; School of Medicine and Health Management, Huazhong University of Science and Technology, Wuhan, China; Dean of School of Medicine and Health Management, Huazhong University of Science and Technology, Wuhan, China

**Keywords:** integrated health care system, prospective payment system, multidisciplinary team, multi-institutional pathway, rural health, China

## Abstract

**Background:**

While integrated health care system has been proved an effective way to help improving patient health and system efficiency, the exact behaviour model and motivation approach are not so clear in poor rural areas where health human resources and continuous service provision are urgently needed. To gather solid evidence, we initiated a comprehensive intervention project in Qianjiang District, southwest part of rural China in 2012. And after one-year's pilot, we developed an intervention package of team service, comprehensive pathway and prospective- and performance-based payment system.

**Methods:**

To testify the potential influence of payment interventions, we use clustered randomised controlled trial, 60 clusters are grouped into two treatment groups and one control group to compare the time and group differences. Difference-in-differences model and structural equation modelling will be used to analyse the intervention effects and pathway. The outcomes are: quality of care, disease burden, supplier cooperative behaviour and patient utilisation behaviour and system efficiency. Repeated multivariate variance analysis will be used to statistically examine the outcome differences.

**Discussion:**

This is the first trial of its kind to prove the effects and efficiency of integrated care. Though we adopted randomised controlled trial to gather the highest rank of evidence, still the fully randomisation was hard to realise in health policy reform experiment. To compensate, the designer should take efforts on control for the potential confounders as much as possible. With this trial, we assume the effects will come from: (1) improvement on the quality of life through risk factors control and lifestyles change on patient's behaviours; (2) improvement on quality of care through continuous care and coordinated supplier behaviours; (3) improvement on the system efficiency through active interaction between suppliers and patients.

**Conclusion:**

The integrated care system needs collaborative work from different levels of caregivers. So it is extremely important to consider the supplier cooperative behaviour. In this trial, we introduced payment system to help the delivery system integration through providing financial incentives to motivate people to play their roles. Also, the multidisciplinary team, the multi-institutional pathway and system global budget and pay-for-performance payment system could afford as a solution.

## Introduction

### Background

Health care delivery must continuously evolve and adapt to the changing context in coordination with the social and economic development, which constantly result in the demographic/epidemiology transition and disease burden shifting. One of the major changes in rural areas these years is the rapid increase of chronic prevalence, causing huge pressure not only on patients but also on the health resources. According to *The Lancet*, it is estimated that “reduction of mortality from cardiovascular disease by only 1% per year between 2010 and 2040 will save 10·7 trillion (US$)” which is “68% of China's real gross domestic product in 2010” [1]. Ever since 1980s, the unique hierarchical “county–township–village” three-tier rural health system has been serving more than 800 million Chinese people. But with the huge growth of chronic demands and shrinking capability of primary care, this system is in danger of falling apart [2]. Integrated care has been proved an effective way to improve health outcomes since the new millennium. Across the European region and other high-income countries, they have long developed numerous practices of initiatives to minimise fragmentation in care delivery and strengthen the quality and continuity of care [3–7]. However, integration reforms have been moving clumsily in low- and middle-income countries, especially in rural areas [8, 9].

### Previous researches

Theoretically, the delivery system needs to be integrated from both horizontal and vertical directions, which refers to not only to integrate primary care and medical service but also to coordinate the suppliers from three levels. Although integrated care attaches importance on patients' interests; however, it is hard to consider patient without integrating the suppliers' interest and bring up a new behaviour mode. As crucial as it is to achieve a “mutually benefit collaboration” as Zhu (2009) suggested [10], Ye (2013) appealed to “integrate the providers' interests in cooperation” before make “a sufficient referral mechanism with different levels of provision” work [11]. On how to integrate the suppliers' behaviour, Korda (2011) pointed out that it rely on “interdisciplinary provider teams to coordinate patient care; health information and other technologies to assure, monitor, and assess quality, and payment and financial incentives such as bundling, pay-for-performance, and gain-sharing to encourage value-based health care” [12]. Also agreed with Korda was Wodarski (2014) who stated that professional integration was important because it had great impact on “patient health behavior change in primary care” [13]. Pati (2011) claimed that the multidisciplinary team (MDT) could help care integration from different institutions [14], and Degaspari's research (2013) proved that MDT could help improving multi-institutional system efficiency [15]. However MDT was more often used in cancer and acute disease treatment [16, 17], few studies can be found on its effectiveness in community-based chronic disease treatment, which is also the case of integrated care pathway (ICP) [18]. But some earlier researchers had found out that ICP had a big influence in non-clinical area, such as serving as a useful communication mechanism to reinforce the organisational coordination (Simkiss, 2005) [19], and contributing to consistent quality improvement through benchmarking (Fowell, 2002) [20].

On the other hand, payment system crucially influences the institutional cooperation behaviour through financial incentive provision. However the dominant fee-for-service payment system in Rural Medical Insurance Scheme plays a role of fragmenting rather than integrating the delivery system. Zhou (2014) criticised that the retrospective payment system should take account for “fast-growing medical costs”, and professional collaboration were “greatly reduced” [21]. However the pre-paid system also has its own weakness in possibly encouraging adverse-selection to reduce the patient visits and thus deteriorate the access equity [22, 23]. Robinson (2004) recommended the “blending payment” to approximately avoid the defects of pure retrospective and prospective payment system [24]. After evaluating the Blue Shield of California Global Budget System (GBS) reform, Markovich (2012) found out that the GBS could help solving adverse motivation by “sharing risk and savings among partners for providing health care” [25]. Though there is no strong evidence so far on how to design the most suitable payment system to stimulate “professional cooperative behaviors” between multi-institutional while bringing no moral harm, as Cunningham (2013) concluded [26], we still believe blending payment system is the right direction. We should carefully design to blend the strengths of different payment systems to activate the greatest efficiency of multi-institutional collaboration and thus to achieve the goal of patient interests.

### Trial introduction

To explore the hidden mechanisms of integrated care, we initiated a multifaceted intervention project in Qianjiang (pronouncing “Chan-jung”) District, a rural county with a modest population size and relatively isolated location in Chongqing Municipalities, southwestern part of China. The whole project was population-based and patient-centred; the intervention targets were basically rural dwellers with chronic diseases (hypertension and/or diabetes II, concurrent situations included). The packaged strategies invented a new behaviours model including MDT and multi-institutional clinical and management pathway (MIP); also we changed the fee-for-service payment system into a “system global budgets and performance-based payment system (SGB–P4P)” to promote the behaviour efficiency. This project aimed to implementing integration across community care and hospital service, which should bring primary caregivers, clinicians, specialists, managing staff together. Especially, this integration was designed to merge over-boarder between care delivery system, public health system and medical insurance payment system, which called for integration not only in health system but also between health sectors.

#### Trial status

The study design had passed a cross-national peer review and accepted grants by the China Medical Board. It was also registered in the Chinese Clinical Trial Registry (ChiCTR-OOR-14005563). The pilot study had been done between July 2012 and December 2012. The main study begun in June 2013 and was designed to run for 24 months. Baseline data were collected in July–August 2012 and the end-line investigation will be done in May 2015. We had just started data cleaning; the final results will come out after May 2015.

## Trial setting

### Geography remoteness and economy weakness

**Figure 1.**
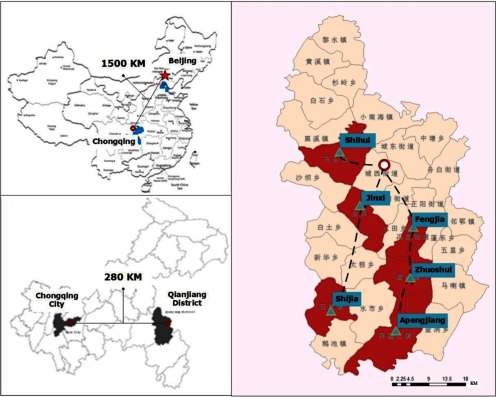
Map of China, Chongqing and Qianjiang: geographic distribution of trial places.

Qianjiang District is a typical rural area locates in southeast Chongqing, the only municipality who sits in the deeper part of southwest China and thus is less developed compared to the other three municipalities. Qianjiang had a population size of 550,000 people whose average income per capita per year in the past five years was under US$480, relatively half of the whole nation's spectrum (data resource: Qianjiang District National Economic and Social Development Statistics Bulletin 2001–2012). Qianjiang has in total 30 communities and 24 of them are rural ones. The average town population size is about 12,000, and every town has around 10 villages. Rural people accounted for 80% of the total population. Over 90% of the rural people have enrolled in the New Rural Cooperative Medical Scheme, which allow them to get nearly 60% (2012) reimbursement of the total in-patient medical expenditure from the medical insurance fund. The unique mountainous terrain causes the average travel time from rural towns to downtown unexpectedly high, varying from 0.5 hour to 6 hours (by mass transportation), walking and transferring time not included (see Figure 1).

### High chronic disease prevalence and poor patient health status

The prevalence of hypertension/diabetes was 17.7%/3.8% in the nearest epidemiological investigation before trial (Qianjiang Animal Disease Surveillance and Epidemiological Investigation, 2012); namely, there were theoretically nearly 3,000 chronic patients in average in each town who has with either hypertension/diabetes (or both). Though Chronic Disease Management Project was rocketed up since the Chinese Government initiated its National Public Health Reform as part of the New National Medical System Reform in 2009, the proportion of patients under standard management was only 30% in 2012. The chronic patient who was under good control and had good compliance of medicine-taking was less than 15%, and the quality of life (QOL) of hypertension/diabetic patients was only 49/44 QALYs, due to our earlier investigation before the trial.

### Human resource shortage

Two major county hospitals and 24 township hospitals took the responsibility of medical treatment for rural chronic patients in this area. There were about 20 doctors/licensed practicing physicians from Departments of Endocrinology, Neurology and Cardiology in each county hospitals, which mainly deal with hypertension, diabetes and the complications such as stroke, coronary heart disease, diabetic foot and diabetic eye diseases. In each township hospital, there were about five medical staff and three primary care staff who provide basic medical treatments and regular follow-ups; in each village, there were one or two doctors who provide simple medical treatments and prescribe basic medicines like Captopril or Nifedipine. In all, the 40 doctors from county hospitals and 8 medical staff from each township hospital took care of over 90% of the primary/secondary/tertiary care for all the chronic patients of its own town. It is not difficult to conclude that, the inadequate health human resource, both in quality and quantity, is one of the major problems that hinder the improvement of quality care.

To sum up, Qianjiang rural area has problems of extremely low availability of access and human resource, thus create terrible consequences as: (1) decreasing quality of care; (2) dropping down of QOL; and (3) raising up of the disease burden. To make the inequity problem even worse, due to inconvenient medical consultancy and reluctance visits, poorer people often get severe complications before they are willing to see a doctor, and less poor people are often out of appropriate control to seek for higher medical treatment. This situation would create the most harmful result of the patient flow disorder and leap of medical expenditures, and also would diminish the original health resource distribution and system efficiency, which we believe is the worst tendency in nowadays rural areas no matter where they come from.

## Methodology

### Study design

A clustered randomised controlled trial was used. Nearly 60 clusters/villages in 6 towns and 200 professionals from three levels were intervened, and around 6000 chronic patients were enrolled in this trial. We re-designed the health policy by giving initials to change their delivery and payment system on chronic diseases. There were two basic intervention modes: (1) care integration (MDT and MIP), which was to integrate different caregivers aiming to reshape the solitaire county–town–village service frame into an integrated health care delivery system; (2) payment integration (SGB–P4P), which was to integrate between the delivery system and medical insurance payment system by alternating the original fee-for-service system into a pre-paid system. This strategy aimed to create incentives to help the suppliers to act more willingly or actively. In accordance, the 60 clusters were categorised into three groups: two treatment groups which implemented each of the above strategy; and one control group with no interventions to serve as a natural blank comparison.

### Hypothesis

**Figure 2.**
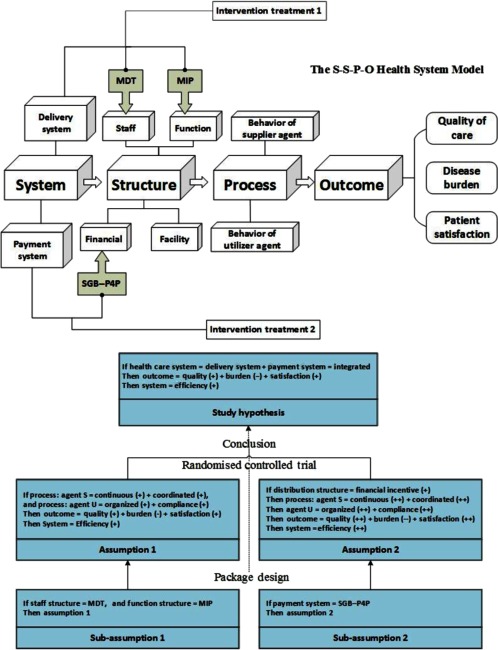
The study hypothesis, assumptions and proof paradigm.

The whole hypothesis was made based on the evolving model of Andersen's service utilisation behaviour model and Donabedian's structure-process-outcome paradigm to assess the health quality [27, 28]. We modified the health system structure referring to some suggested modification to adapt to the chronic cases [29–31]. We made two basic assumptions under the transduction pathways from hypothesis to conclusion: (1) the integrated health service delivery would improve health outcomes through providing more coordinated care, and the system efficiency would then improve from better primary and community care; (2) the payment system would help to change the supplier behaviours and exemplify the outcomes. Assumption 1 would be proved from comparing treatment groups with the control group; and Assumption 2 would be proved from comparison within this two treatment groups (see Figure 2).

### Intervention strategies

Based on the literature analysis, we assumed the key factors behind the discontinuity of care were: lack of human resource, lack of standardised cooperative behaviour model and lack of financial incentives to motivate professional collaboration. Therefore, the breakthroughs were needed at least in these three aspects: (1) the effective form to organise health staff; (2) the behaviour model to instruct cooperation; (3) the financial incentives to stimulate professionals and organisations to collaborate. Accordingly, we developed the intervention packages which contained: (1) MDT, the multidisciplinary team service model to re-organise the health staff; (2) MIP, the multi-institutional clinical and management pathway to re-enact the cooperative behaviour; and (3) SGB–P4P – the system global budgets and performance-based payment system to re-distribute the financial incentives. The first 2 strategies were linkage mechanisms to integrate care between health professionals from different organisations and levels; the last strategy was a motivator which would potentially affect the supplier behaviours by providing shared-interests and goals (see Table 1).

#### Multidisciplinary team service

**Figure 3.**
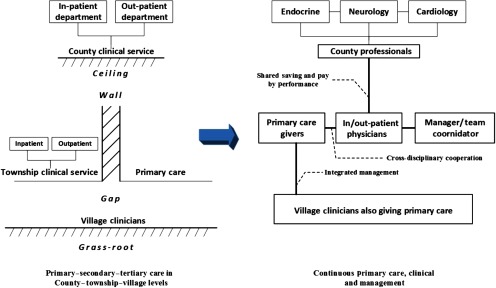
MDT: re-construction of the health human resource in three levels.

The MDT was developed to help both the primary and hospital care and suppliers integrate under the situation of scarce human resources. Considering the unique county–township–village human resource distribution structure in rural area, we designed a “double L” team – a unique structure to connect three service levels from both horizontal and vertical direction. The upper vertical ladder connected the clinical physicians between county–township level, and the lower vertical ladder connected the primary caregivers between township–village level; and the interphase was the connection of clinical and primary staff through coordination management in township level. In this trial, we enrolled in 15 doctors in each county hospital, 2 from out-patient and 3 from in-patient Departments of Endocrinology, Neurology and Cardiology, to take care of the continuous treatment and contact with the community doctors. In every intervened town we enrolled six staffs: two physicians, one responsible for out-patient and one for in-patient; three primary caregivers, mainly took care of screening, following up and monitoring the chronic patients; and also one team manager/coordinator, to help the team communication and information delivery. In every cluster of the intervened town we enrolled all the village clinicians to help the neighbourhood follow-up. By this means we formed a MDT from different disciplines as primary care, clinical service and management, and also from different organisations as county hospital, township hospital and village clinics; and also made sure there to be at least 17 clinicians, 10 primary caregivers and 4 management staff in every community to serve the chronic patients (see Figure 3).

In order to bring these people together and act for the patient good, we set up several mechanisms to shorten the distance between layers. *Shared information*: patient information like medical record, health documents, images, continued treatment suggestions and informed consent could be shared between different suppliers when patients are doing referrals or in any other necessary cases. *Two-stage group learning*: every month, the county doctors would go to township hospitals to give lectures on how to diagnose and treat hypertension/diabetic patients to clinicians, to review the last month discharged patients (or else they had to travel long to have their own) and to have a case discussion with the local staff. Then the township doctors would consequently give lectures and discuss cases with the village doctors. The two-stage learning procedure was proved to be much better than having a large crowd with different education background in the same room. Also, the group learning must be recorded by the team manager and finally assessed by the project officer. *Short-time training*: every season, the team manager would send one or two staff to the county hospital to have a short-time training lasting for about two weeks, making quick knowledge/skill absorption on what he/she mostly need.

#### Multi-institutional clinical and management pathway

Once we built the MDT, we also need a new behaviour mode to instruct the team cooperation. To connect the primary–secondary–tertiary care among community and hospital level, we changed the original segmented practice into a continuous disease-based pathway to integrated care and management comprehensively and jointed efforts from the three levels. The aim was to merge all the services such as screening, diagnosis, treatment, rehabilitation and community management together into a continuous and coordinated care cycle through specific disease pathways and flow management. The pathway composed three parts: (1) the regular community practice provision under stable condition, including screening, BP/risk factor monitoring and follow-up of rehabilitation patients; (2) the continuous clinical treatment under incidence, including medical condition assessment, continuous treatment, variance analysis and rehabilitation; (3) the flow management, including professional contact, referral, file transfer and joint follow-up. Through backstage management tools we connected all the professionals' behaviours into a conjoint medical process by removing what was superfluous and filling in what was missing under the original context.

**Figure 4.**
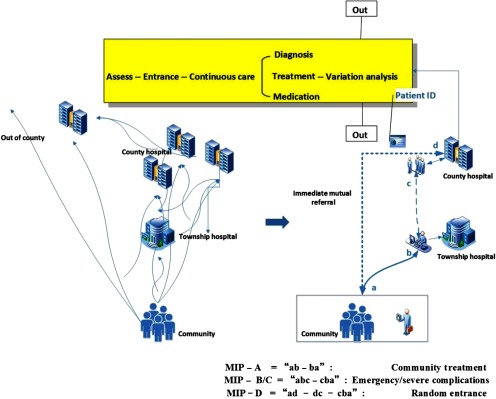
MIP: re-enactment of the organisation collaboration and patient flow.

There were four types of pathway. A-type was community treatment, treating patients in the village and township levels. Under this pathway, the patients with minor occasions might be cured by simple medication. Once the patients finished visits, he/she would just turn back home and there would be an in-door follow-up offered by the nearest village doctor within three days to make sure if he/she had recovered, if not, the management level would be upgraded and the patient would be transferred to a higher-level institution. This would save the inconvenience for the remote and old residents. B-type was emergency case, and C-type was complex/serious complications. Under either of these circumstances, once the village/township doctors found out such cases they had to contact with the corresponding county coordinator and transfer the patients to the relevant department. The cases were defined as: (1) instant BP ≥ 180/120 mm Hg/GLU ≥ 16.7 m mol/L; (2) severely damaged target organ; (3) and/or had other complications from cardiovascular, cerebrovascular and circulatory system. During the hospitalisation period, they should re-contact the county doctor to track the patients' status and follow-up after the patients were discharged. D-type was random entrance, including patients who were newly recognised or had a random entrance from outside of the pathway. Whenever it happened, the patients would be identified by their ID card and enrolled in MIP, and the doctor who discharged the patient must contact his/her neighbourhood doctor to establish a new file in the first place and start to follow-up (see Figure 4).

We made specific guidelines on how to assess and recruit the patient into the MIP based on complication diseases: coronary heart disease, stable angina, unstable angina, ST elevation acute myocardial infarction, non-ST elevation acute myocardial infarction, heart failure, arrhythmia, ketoacidosis, non-ketotic hyperosmolar syndrome, lactic acidosis, diabetic nephropathy, diabetic patients with cerebrovascular disease, diabetic ketoacidosis diabetic coma. In these pathways we pacifically pointed out what measurement the township physician should provide and what the county professionals should/should not do based on the first stage treatment.

#### System global budgets and pay-for-performance (SGB and P4P)

Though MIP and MDT were designed to form a new patient-centred service model, the behind motivation to make it run effectively was still needed. Originally, the suppliers were paid separately from the medical insurance fund without considering if they cooperate, in this case, they would be granted reimbursement whether they performed duplicated exams, prescribed contradictory medicine or had bad control of discharged patients. The whole concept of SGB–P4P system was to better integrate the primary care fund and medical insurance fund together, and to stimulate team staff from different institutions to act for the patient interest. The basic process was: first re-calculating the possible medical consumption per capita in a certain period, and pre-paying the MDT team as a whole without identifying where they came from; second paying based on the cooperation performance; third promising the re-distribution autonomy of the possible savings and paying according to the previous distribution plan. The medical fund officer kept the right to evaluate the team performance, monitor the money re-distribution plan and randomly check to make sure the patients were treated properly. The logic behind the payment system was to encourage the dominant attention on clinical service gradually transfer into the primary care, and avoid inappropriate and unnecessary treatment to the greatest extent by integrating the suppliers into a shared group and force them to think and act for the patient good as much as possible.

To sum up, the MIP was designed to afford a behaviour benchmark, the MDT was designed to distribute role responsibility, and the SGB–P4P was designed to make it effectively and consistently run.

### Sample size and power calculation

Bound by the limited resources, we took several steps to decide the sample size to simplify the management while promise the scientifically meaningful differences. We used Power Analysis and Sample Size (PASS, 11.0) to help us calculate the statistical power and sample size. A statistical power of 80% with 0.05 significance level was chosen.

(1) *Cases of continuity of clinical care*. First, we used *two independent proportion calculation* to estimate the continuous cases needed in terms of *continuity of clinical service* (see “Outcome”), which would mostly constrain the statistical power. The results showed that although 100 cases in the treatment group could promise a required power, with the uncertainty of sample size in control group, we needed at least 120 cases in the treatment group to detect a 20% difference between groups with required significance level (see Table 2). (2) *Sample size*. We decided the minimum sample size based on the knowledge of cases needed and previous data. According to the previous reimbursement records in trial place (2010–2012), the continuous cases accounted for 3.0–5.0% among the chronic hypertensive patients, so with a conservative consideration, we need at least 230–270 individuals per town (530/490 per group) to ensure that we could have 120 continuous cases in each group. (3) *Power calculation*. Third, according to the estimated sample size we re-calculated the statistical powers under other indexes to check out whether that the sample size was sufficient. We chose several main variables and analysis methods to calculate the possible statistical power and output the required odds ratios (see Table 3). (4) *Drop-outs*. Taking a conservative drop-out rate of 20%, we finally arrived at an estimated sample size of 258–324 per town.

### Sampling and randomisation

Six towns were selected randomly from the 24 rural communities and then were categorised into three groups with a combined consideration of population size, social development and distance. Because there were obvious potential confounders in social experiment, the towns were first categorised into two types based on comprehensive consideration of socio-economic development level and hospital ability, two basic confounders which we assumed high relevance with the intervention outcome. The socio-economic development level mainly indicated the patient population, their per capita income and average distance from the location to downtown (translated into traffic time by public transportation). The hospital ability represented resource possession and staff skills. Second, every two towns of each type were randomly assigned into treatment/control groups to ensure group comparability. These two steps were done to make sure towns comparable both within groups and within cluster types in case of different comparison need.

In patient sampling, we used three-stage stratified random sampling strategy instead of propensity scoring matching, concerning the complexity of sampling and excessive workload. We chose gender, age and risk level as the three stratify factors. Risk level was a comprehensive factor that summing up the BP/GLU level as well as the possible risky factors due to the patient lifestyle. The hypertension and diabetes II patients were divided into three and two risking groups before the baseline, and we stratified the patient library mainly in accordance with the original files but with a slight alternation based on professional re-assessment after personal contact at the baseline.

#### The inclusion criteria

The inclusion criteria of participants for investigation and following-up were patients who: (1) had been registered as a managed chronic patient between year 2008 to January 2012, which meant the participants were all aged over 35 and had an official health records including their basic demographic information, symptoms and risk factors, and with a history of hypertension and/or diabetes II no less than six months and taking BP/GLU records at least four times a year; (2) had been consistently enrolled in the new rural cooperative medical scheme; (3) constantly reside in his/her own cluster, which was defined for at least one year before intervention and at least six months after and must ensure that have a complete BP record.

#### The exclusion criteria

The exclusion criteria were those who: (1) had a stable BP/GLU history (consistently under 120/80 mm Hg and 6.5 m mol/L) longer than one year and thus would not admit having chronic diseases or refuse to take medicines; (2) were estimated for a life expectancy less than two years due to old age, venerable situations or severe complications such as cerebral infarction or pancreatic cancer, thus would potentially call for apparent extra samples; (3) would probably get lost in follow-ups with high chances to go out, which was recognised for at least six months away yearly during the intervention period for reasons as migration for work, education or kinship-care-seeking; (4) would hardly visit or investigate due to intellectual or activity incompetence; (5) mentally damaged or communication incapable; (6) refuse to cooperate due to personal reasons. Newly recorded patients between intervention period were not in our sample giving that they would mostly be managed under risk level I and unnecessarily shake the sample structure.

Finally 1641 patients with hypertension and 332 with diabetes II of the total 6833 managed subjects were selected from the record list provided by the local administrator (see Table 4). At the baseline and end-line of this trial, we will take all the samples into statistical examination, checking out if there were any differences.

### Enrollment and masking

A random sequence of numbers was generated by the computer under each stratify factors. Patient enrollment and investigation were personally done by 12 investigators trained by our research team. All the respondents were irreplaceable samples, meaning that they would be traced through the whole intervention period and accept investigations both in the baseline and end-line study. During intervention, the missing respondents would not be replaced by matching substitution samples, and newly screened out patients during intervention period would not be considered. Due to the trial design of provider intervention, the masking was not feasible, but the patients were not informed with the grouping information and they were not aware of what change would be given to care supply. When trial finished, we will send the database to the third player to do the analysis without giving them the group intervention information.

### Ethical approval

This trial has received ethnical approval from the Tongji Medical College Academic Ethics Committee of Huazhong University of Science and Technology (IORG No: IORG0003571) in June 2012. Patient informed consents were achieved in the baseline investigation.

### Outcomes measures

Basically, we take the period BP/GLU control rate and drug compliance (DC) as the primary outcomes. Period BP/GLU control rate is a new index we create to reflect the consistent BP/GLU control in a certain period, to replace the old index which merely consider if the BP/GLU is safe by the once time assessment. Period BP/GLU control rate takes the fluctuation degree and frequency into account; it changes reversely with the frequency and degree of BP ≥ 140/90 mm Hg (130/80 mm Hg with complications)/GLU ≥ 7.8 m mol/L (after meal) within the total times of BP/GLU assessment. The *BP control rate* is defined as the ratio of systolic pressure under 140 mm Hg (130 mm Hg with complications) among the sample (N), and CT means control times.
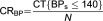


For the population, we use the arithmetic mean of total individual period BP/GLU control rates to evaluate the group control rate.

*DC rate* is defines as the ratio of good DC among the sample which is measured by the Morisky questionnaire. The BPs are collected at least every three months during the intervention, and the DCs are collected twice at the baseline and end-line investigation.

#### Outcome measures: “quality of care” & “disease burden”

Quality of care refers to: (1) control rate, including the average personal BP/GLU control scores and population BP/GLU control rate; (2) cases of new complications per capita during intervention period; (3) re-admission rate within 30 days; (4) quality of treatment, including recovery rate, consistency rate of admission–and–discharge diagnosis, consistency rate of township–and–county hospital diagnosis, consistency of medication; (5) patient satisfaction rate.

Disease burden refers to: (1) QOL was measured by the Short Form Health Survey Questionnaire (SF-36)”; (2) average medication burden per chronic capita, measured by Standardized Drug Price Investigation.

#### Process measures: “patient behaviour” and “supplier behaviour”

Patient behaviours refer to: (1) population compliance rate, including compliance to medication and compliance to follow-ups; (2) lifestyle change rate, including less salt/fat/cigarette/alcohol, more exercise and stable emotion control; (3) patient awareness rate, including awareness of risk factors, complications and other seven questions of chronic disease knowledge; (4) self-efficacy rate, including patient efficacy of control their own behaviours.

Supplier behaviours refer to: (1) referrals, including emergency referrals, regular referrals and rehabilitation referrals; (2) communication intensity, including communication frequency between different clinicians and between clinician-and-primary caregivers; (3) the cases of unnecessary exams per capita; (4) the average referral time and waiting time; (4) staff satisfaction scores.

Besides, the index of continuous service rate refers to patient–and–supplier behaviour, which indicates the continuous contact of the same patient and primary caregivers. We use continuity of care index (COCI) to reflect the continuous contact of the same patient and primary caregivers, which is important index examining the possibility of high quality in primary care provision [32]:
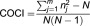


In this calculation, *N* stands for the sample size, and *n* stands for different providers' number; and COCI reflects the dispersion of personal contacts. The COCI takes on values between 0 and 1. We assume that the continuous contact with the same primary caregivers will do most good for our patients, so a value of 1 signifies the situation when the same provider is seen at every visit, and 0 signifies when different providers are seen.

#### Structure measures: “system efficiency”

The system efficiency mostly refers to the compare relation of medical expenditure of county–to–township hospitals, including inpatient visits, in-patient fee, average length of stay, average cost per case, average exams per case and the average use of necessary medicine. And integration degree of MDT is measured by the Integration Degree Questionnaire evolved from D'Amour's collaboration typology model [33].

#### Project costing

To make an economic evaluation of the policy trial to compare with the usual care model, we also take records of the extra expenditures of project such as publicity, training, patient ID card production, printing, transportation, meeting organisation, consumables and accommodation. Besides, the increased staff workload is also recorded into counting the cost by adding the working hours into cash under the National Labor Payment Standard and the average local salary. The team coordinators were asked to take a monthly record of all the actual expenditures and increased working hours.

All data were collected through structured patient questionnaire, staff questionnaire, institution investigation, follow-up files, medical records and the reimbursement data that extracted from the local electronic insurance system (see Table 5).

### Analysis strategy

#### Statistical analysis

There are two-stage analyses in this trial. First, we will use difference-in-differences (DID) model [34] to examine the effects of the intervention; second we will use structural equation modelling (SEM) [35] to explore the hidden effecting pathway of how the intervention works.

The DID model is often used to exclude out the time effect and group differences. In the pilot study, we had mimicked the regression tendency of BP falling to ensure a similar time effect in groups. In the main study, we mainly include three explanatory variables in the regression model: group, time and interaction term between group and time. And to exclude the intra-cluster correlation (ICC) from non-independent clustering and cross-level interaction, we choose three-level regression model to control for the potential confounders of the context effect. For other repeatedly measured outcomes like BP control, lifestyle changes and professional behaviours we mainly use three-level and repeated measure multivariate analysis of variance to examine the group differences. The three levels we consider are: level 1 – individual, level 2 – cluster/village, level 3 – group. Data analysis will be carried out on the Stata (IC: 13.1). In SEM we hope to establish an explainable relationship between intervention effect of health outcome and design effect of payment system reform.

#### Process evaluation

We first use contextual analysis to describe the fundamental environment of the delivery system in the trial place, and then demonstrate the intervention process on organising MDTs, MIPs and implementing SGB–P4P. We will especially focus on the possible obstacles which would potentially hinder the implementation of intervention strategy. Then we will mainly discuss the effecting pathway of payment system to health outcomes, and emphasise on key elements of behaviour model from both suppliers and utilisers side. At last by following cause–effect paradigm, we use evidence to prove our assumption and hypothesis. Relative resources and materials are collected from document files, behaviour observations and stakeholder interviews; data analysis will be processed using structured narrative analysis.

## Discussion

This is the first trial in both developing countries and rural areas to gather evidence of health policy reform on delivery and payment system integration using experimental design. Previous studies on chronic disease management mostly focused on clinical and technical novelties, and did not develop inter-systems vision and strategy when reforming the delivery system [36, 37]. Although there were researches which had already proved the importance of simultaneous payment system intervention on changing delivery system [38–40], we still do not know in what way and to what extent can it affect the health outcomes. Thus, through this trail we first hope to build a feasible policy package to help health system integration, and then we want to identify the barriers and facilitators during implementation of professional/organisational collaboration on community chronic disease management. By doing this, we especially expect to prove the importance of integrated and systematic thinking when making decisions of health policies. The outputs will be an integrated care model of community-based chronic disease control, including policy packages of clinical guidelines, team guideline and financial stimulation plan. With the fundamental environment analysis we hope this model can be generalised to or at least give some hints to other LMICs/rural areas which are facing with similar problems.

The trial design has some potential limitations. Initially, we were going to use randomised controlled trial which may provide the highest level of evidence even in social science experiment as many previous studies in China succeeded to [41–43]. However, the intervention strategies we designed in this trial will not succeed without the acknowledgement in each cluster, which means the randomisation process might sacrifice a little. As a result, the proof power may be reduced. To compensate, we controlled the potential contextual and individual confounders during the grouping and sampling period to achieve a maximum control of cases. Second, though a latest systematic review pointed out that there was no evidence to prove that the multifaceted interventions were more effective in changing the health care professionals' behaviours [44], still several experiments had shown better effects of multifaceted interventions on hypertension and diabetes II patients [45–47].

## Conclusion

As it is a strong belief that “the real change in all kinds of reforms is the change of people”, it is extremely attached importance to the professional collaboration in the health integration reforms area. With various theory and practice of nowadays integration reforms, we choose the integration between primary care and clinical treatment in delivery system, and designed this trial based on community chronic patient, exploring the possibility of cooperation behaviour among multidisciplinary and multi-institutions. With further data analysis, we will provide evidence not only on evaluation of the impact of interventions to improve the integrated care, but also evaluation of the effects and efficiency of improved integration.

## Figures and Tables

**Table 1.**
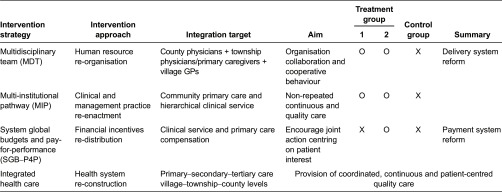
Group intervention strategies

**Table 2.**
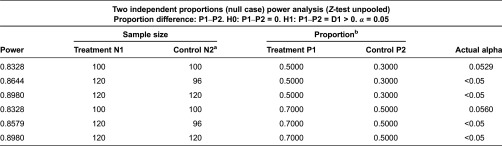
Minimum cases needed under index “continuity of clinical service”

Summary: group cases of 120/96 or 120/120 in treatment/control group could achieve over 80% power to detect a 0.2000 difference between group proportions.

^a^Considering the intervention effect, we assumed the relation of conforming cases between control/treatment group varied from 0.8–1.0/1.0.

^b^Assumed the detectable proportion of cases conformed to the defined “continuity of clinical service” is 0.5–0.7 in the treatment group and 0.3–0.5 in the control group.

**Table 3.**
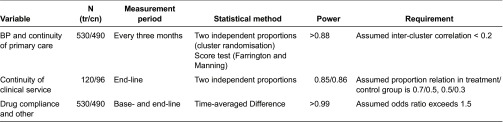
Possible statistical power of main variables

**Table 4.**
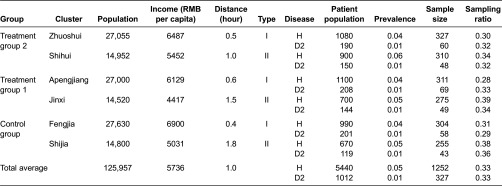
Result of grouping and patient sampling

Note: ① Type A for better cluster: larger and richer; type B for worse cluster: smaller and poorer. ② H for hypertension; D2 for diabetes 2. ③ Population, patient population and sample size are not averaged under “total average”. ④ All variables are compared with treatment group 1. ⑤ Distance is transferred into traffic time by public transportation. Time varies in accordance to the cluster terrain instead of linear map distance.

**Table 5.**
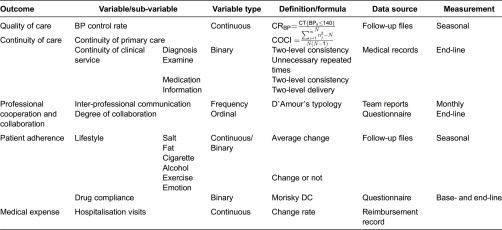
Outcomes, definition and data collection
